# Gene-Set Enrichment Analysis for Identifying Genes and Biological Activities Associated with Growth Traits in Dromedaries

**DOI:** 10.3390/ani12020184

**Published:** 2022-01-13

**Authors:** Morteza Bitaraf Sani, Zahra Roudbari, Omid Karimi, Mohammad Hossein Banabazi, Saeid Esmaeilkhanian, Nader Asadzadeh, Javad Zare Harofte, Ali Shafei Naderi, Pamela Anna Burger

**Affiliations:** 1 Animal Science Research Department, Yazd Agricultural and Natural Resources Research and Education Center, Agricultural Research, Education & Extension Organization (AREEO), Yazd 8915813155, Iran; javadzare49@gmail.com (J.Z.H.); ashnaderi@gmail.com (A.S.N.); 2 Department of Animal Science, Faculty of Agriculture, University of Jiroft, Jiroft 7867155311, Iran; rodbari.zahra@gmail.com; 3 Department of Animal Viral Diseases Research, Razi Vaccine and Serum Research Institute, Agricultural Research, Education and Extension Organization, Karaj 3146618361, Iran; o.karimi@areeo.ac.ir; 4 Animal Science Research Institute of Iran, Agricultural Research, Education and Extension Organization (AREEO), Karaj 3146618361, Iran; hossein.banabazi@gmail.com (M.H.B.); s.smailkhanian@areeo.ac.ir (S.E.); naderasadzadeh4@gmail.com (N.A.); 5 Department of Animal Breeding and Genetics (HGEN), Centre for Veterinary Medicine and Animal Science (VHC), Swedish University of Agricultural Sciences (SLU), 75007 Uppsala, Sweden; 6 Research Institute of Wildlife Ecology, Vetmeduni Vienna, 1160 Vienna, Austria; pamela.burger@vetmeduni.ac.at

**Keywords:** growth, biological theme, gene ontology, dromedaries

## Abstract

**Simple Summary:**

This project aimed to find biological themes affecting growth in dromedaries. Candidate SNPs associated with growth were mapped to 22 genes, and 25 significant themes were identified related to growth. The main biological functions included calcium ion binding, protein binding, DNA-binding transcription factor activity, protein kinase activity, tropomyosin binding, myosin complex, actin-binding, ATP binding, receptor signaling pathway via *JAK-STAT*, and cytokine activity. *EFCAB5*, *MTIF2*, *MYO3A*, *TBX15*, *IFNL3*, *PREX1*, and *TMOD3* genes are candidates for improving growth in camel breeding programs.

**Abstract:**

Growth is an important heritable economic trait for dromedaries and necessary for planning a successful breeding program. Until now, genome-wide association studies (GWAS) and QTL-mapping have identified significant single nucleotide polymorphisms (SNPs) associated with growth in domestic animals, but in dromedaries, the number of studies is very low. This project aimed to find biological themes affecting growth in dromedaries. In the first step, 99 candidate SNPs were chosen from a previously established set of SNPs associated with body weight, gain, and birth weight in Iranian dromedaries. Next, 0.5 kb upstream and downstream of each candidate SNP were selected from NCBI (assembly accession: GCA_000803125.3). The annotation of fragments with candidate SNPs regarding the reference genome was retrieved using the Blast2GO tool. Candidate SNPs associated with growth were mapped to 22 genes, and 25 significant biological themes were identified to be related to growth in dromedaries. The main biological functions included calcium ion binding, protein binding, DNA-binding transcription factor activity, protein kinase activity, tropomyosin binding, myosin complex, actin-binding, ATP binding, receptor signaling pathway via *JAK-STAT*, and cytokine activity. *EFCAB5*, *MTIF2*, *MYO3A*, *TBX15*, *IFNL3*, *PREX1*, and *TMOD3* genes are candidates for improving growth in camel breeding programs.

## 1. Introduction

Understanding genetic factors that influence animal growth is very important for accurate selection and genetic improvement of these quantitative traits. Growth traits, especially birth and weaning weight and gain per day, are economically important heritable traits and necessary for planning a successful camel breeding program [1,2]. Vice versa, good knowledge about genomic pathways underlying a breeding program is necessary to optimize the growth of animals [3]. Many SNPs associated with growth have been detected using GWAS and QTL mapping in livestock [4]; however, few projects have been performed yet for the genetic improvement of growth and meat production in camels.

Approximately 3,000,000 camels are slaughtered annually, and camel meat production reaches 653,000 tons (FAO 2021), although contributing approximately only 0.18% to total red meat production. There are approximately 140,000 dromedaries in Iran (FAO, 2019) that are four basic types, milk, meat, dual purpose, and riding camels [5]. The majority of dromedaries in Iran’s central desert belong to the meat type, which is characterized by a heavy and large head, short neck, wide posterior pars, and firm body [6]. Most dromedary herds are kept semi-extensive or extensive and are located around the desert or near a village. The owners usually gather calves in spring for sale and fattening. The herd sizes range from 4–5 to 100–150 heads [1]. 

In recent GWAS using genotyping-by-sequencing, Bitaraf Sani et al. [1] identified 99 SNPs potentially associated with important growth traits to improve camel breeding, namely birth weight, daily gain, and body weight. Taking advantage of the comprehensive dataset, here, we aimed to find biological themes and genes affecting growth in dromedaries using a previously established set of associated SNPs.

## 2. Materials and Methods

Biological themes that related to growth were identified using gene-set enrichment analysis. In a previous study, we collected data on 51 herds of dromedaries in five regions of the central desert of Iran in 2018. Among the registered 964 calving between January to May, we genotyped 96 calves and collected 252 body weight records in different times. The samples were genotyped-by-sequencing using two restriction enzymes combination, EcoR1 and HinF1, and paired-end (150 bp) sequencing (10 X) on the Illumina HiSeq 2000. Among 14,522 markers, a total of 99 SNPs were associated with growth traits, and twelve haplotype blocks and 80 tag SNPs were predicted. The accuracy of GEBVs based on the 99 associated SNPs was 0.62, 0.82, and 0.57 for birth weight, daily gain, and body weight. The 99 associated markers with body weight, gain, and birth weight in dromedaries were included as candidate SNPs [1]. Next, 0.5 kb upstream and downstream of each candidate SNP were selected from NCBI. The fragments were aligned to the dromedary reference genome (assembly accession: GCA_000803125.3) using Blast2GO OmicsBox (version 1.4.11), including all (non)coding and pseudo genes [7]. Only genes with at least one significant SNP (Blast Expectation Value = 0.001) were considered significant genes. We identified the functional classes of the genes based on five databases, including GO (http://www.geneontology.org; accessed on 10 January 2022), KEGG (http://www.genome.jp/kegg; accessed on 10 January 2022), Panther (http://www.pantherdb.org; accessed on 10 January 2022), Wikipathway (http://www.Wikipathway.org; accessed on 10 January 2022), and Reactome (http://www.reactome.org; accessed on 10 January 2022). Genes located in the same functional class were considered a group of genes with some special and common features, such as participation in three ontological processes, including biological processes, molecular function, and cellular components. Finally, significant functional classes related to growth traits were tested using hyper geometric distribution and Fisher’s exact test by the software package goseq [8].

## 3. Results and Discussion

**Figure 1:**
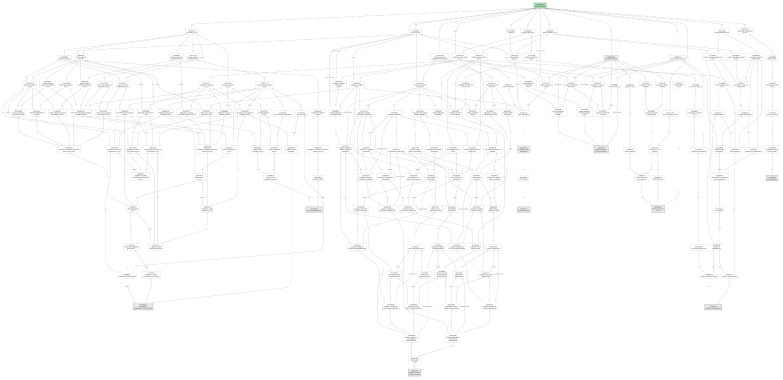
Gene ontology of biology processes for growth traits in dromedaries.

**Figure 2:**
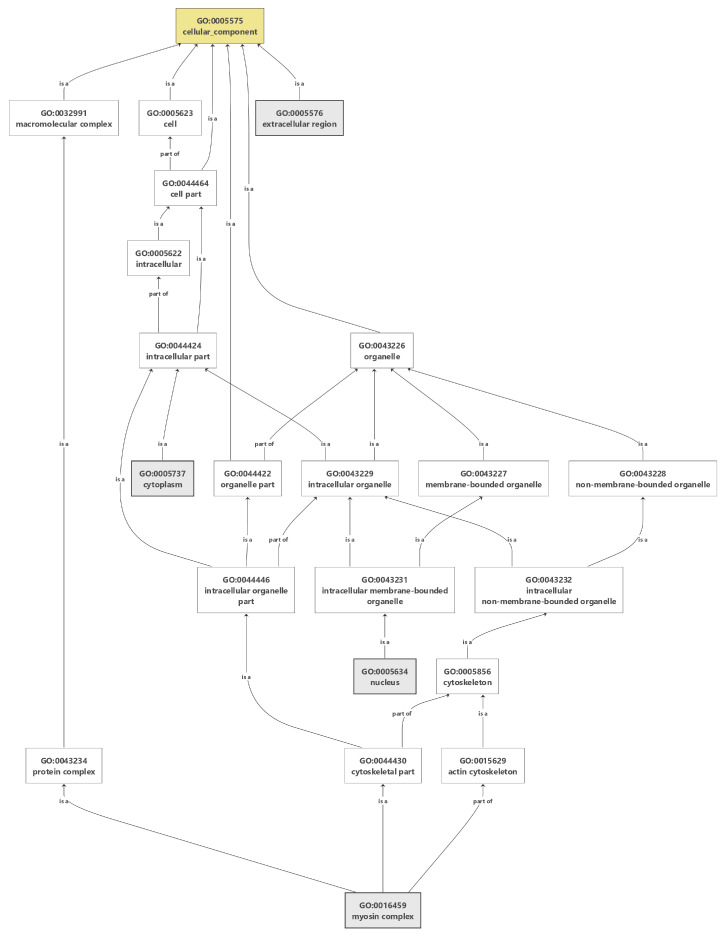
Gene ontology of cellular components for growth traits in dromedaries.

**Figure 3:**
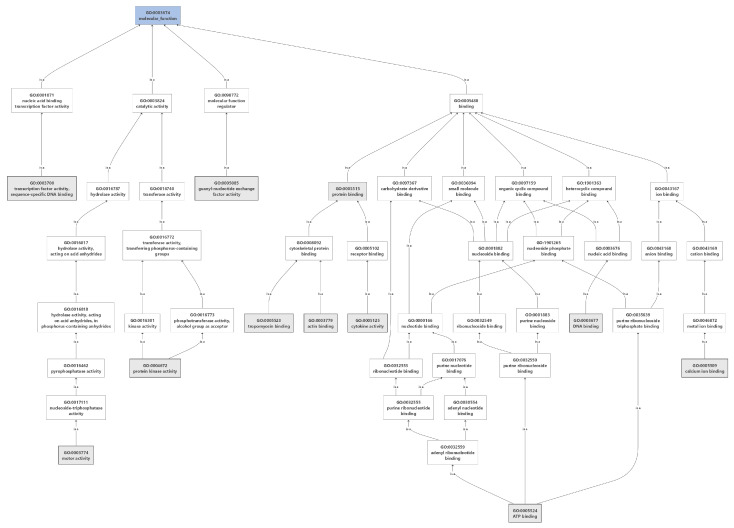
Gene ontology of molecular function for growth traits in dromedaries.

This study aimed to understand biological themes underlying important growth-related traits in dromedaries. We identified 22 genes involved in 25 functional themes (Table 1). Calcium ion binding, including the *EFCAB5* gene, was the most significant terms (e-value = 4.74 × 10^−96^) related to growth. The other main significant terms were DNA-binding transcription factor activity (e-value = 1.13 × 10^−40^), protein kinase activity, tropomyosin binding, myosin complex, actin-binding (e-value = 5.34 × 10^−24^), ATP binding (e-value = 5.34 × 10^−24^), receptor signaling pathway via *JAK-STAT*, and cytokine activity (e-value = 2.93 × 10^−42^), which are enriched by the *MTIF2*, *MYO3A*, *TBX15*, *IFNL3*, *PREX1*, and *TMOD3* genes. The seven biological processes related to growth were detected, including protein phosphorylation; regulation of transcription DNA-templated, protein-end actin filament capping; defense response to the virus; intercellular signal transduction; and response to stimulus (Figure 1). There were four cellular components, including extracellular region, cytoplasm, myosin complex, and nucleus (Figure 2). The 11 significant molecular functions were calcium ion binding, protein binding, cytokine activity, actin-binding, protein kinase activity, ATP binding, guanyl-nucleotide exchange factor activity, DNA-binding transcription factor activity, tropomyosin binding, and motor activity (Figure 3). One of the important genes in these terms was *MYO3A* in the myosin gene superfamily in muscular-skeletal cells [9]. This gene is a protein that plays a major role in muscle regulation and as a transcription factor that directly regulates the expression and differentiation of muscle cells. It is also a regulatory factor in the myogenesis process. *MYO3A* gene has a functional relationship with body mass index [10]. The *EFCAB5* gene is part of the calcium ion binding pathway and is associated with body height and bone density [11]. All muscle fibers use Ca2+ as their main regulatory and signaling molecule. *IFNL3* was another gene related to growth in dromedaries in this study. The interferons (*IFNs*) are classic antiviral cytokines that create the first defense line against infection using antiviral, antitumor, and immunomodulatory activities [12]. The *PREX1* (Phosphatidylinositol-3,4,5-Trisphosphate Dependent Rac Exchange Factor 1) gene involved in the guanyl-nucleotide exchange factor activity pathway encodes a protein that is bonded with guanosine diphosphate (GDP) to release guanosine triphosphate (GTP) and regulate the cytoskeletal structure, gene expression, and reactive oxygen species (ROS) production. Moreover, PREX1 regulates neutrophil responses [13] and plays a key role in actin cytoskeletal rearrangement, adhesion, and the production of reactive oxygen species (ROS) [14]. Additionally, the activity of *PREX1* protein correlates with the induction of actin-mediated membrane ruffling. *Tbx15*, a T-box transcription factor, is to be involved in the regulation of transcription, DNA-templated, DNA binding, DNA-binding transcription factor activity, and nucleus pathways. *Tbx15* has an important role in developing the skeleton, vertebral column, and head by controlling the number of mesenchymal precursor cells and chondrocytes [15]. Moreover, it affects the development of different tissues and organs [16]. *TMOD2* affects growth trait through erythrocyte development, pointed-end actin filament capping, and tropomyosin binding pathways [17].

## 4. Conclusions

This report has identified some terms related to growth in dromedaries using gene-set enrichment analysis. The main significant biological activities were calcium ion binding, protein binding, DNA-binding transcription factor activity, protein kinase activity, tropomyosin binding, myosin complex, actin-binding, ATP binding, receptor signaling pathway via JAK-STAT, and cytokine activity, which can be helpful in improving the understanding of mechanisms of growth. *EFCAB5, MTIF2, MYO3A, TBX15, IFNL3, PREX1*, and *TMOD3* genes are candidates for improving growth in dromedaries. These results provide some insights into camel breeding programs.

## Figures and Tables

**Table 1:** Gene set enrichment analysis significantly (*p* < 0.001) associated with growth traits in dromedaries.

SNP List	Description/Gene Symbol	e-Value	sim mean	GO IDs	GO Names
Chr16:34483240	*EFCAB5*	4.74 × 10^−^^96^	92.67	F: GO:0005509	F: calcium ion binding
Chr14:30865111	endonuclease/reverse transcriptase	5.76 × 10^−^^56^	61.08	F: GO:0005515	F: protein binding
Chr14:30865085	endonuclease/reverse transcriptase	6.36 × 10^−^^56^	61.1	F: GO:0005515	F: protein binding
Chr17:75949	*MTIF2*	7.48 × 10^−^^56^	89.27		
Chr14:30865065	endonuclease/reverse transcriptase	1.52 × 10^−^^53^	61.53	F: GO:0005515	F: protein binding
Chr19:10237661	*IFNL3*	2.93 × 10^−^^42^	92.11	P: GO:0007259; P: GO:0050778; P: GO:0051607; F: GO:0005125; C: GO:0005576	P: receptor signaling pathway via JAK-STAT;
					P: positive regulation of immune response;
					P: defense response to virus;
					F: cytokine activity;
					C: extracellular region
Chr9:22550930	*TBX15*	1.13 × 10^−^^40^	97.45	P: GO:0006355; F: GO:0003677; F: GO:0003700; C: GO:0005634	P: regulation of transcription, DNA-templated;
					F: DNA binding;
					F: DNA-binding transcription factor activity;
					C: nucleus
Chr19:10237641	*IFNL1*	4.46 × 10^−^^38^	93.51	P: GO:0007259; P: GO:0050778; P: GO:0051607; F: GO:0005125; C: GO:0005576	P: receptor signaling pathway via JAK-STAT;
					P: positive regulation of immune response;
					P: defense response to virus; F: cytokine activity;
					C: extracellular region
Chr19:9631630	*LORF2*	1.19 × 10^−^^31^	61.66		
Chr6:14654249	*TMOD3*	2.67 × 10^−^^27^	100	P: GO:0048821; P: GO:0051694; F: GO:0005523	P: erythrocyte development;
					P: pointed-end actin filament capping;
					F: tropomyosin binding
Chr35:9290500	*myo3a*	5.34 × 10^−^^24^	94.62	P: GO:0006468;	P: protein phosphorylation;
					P: visual perception;
					P: response to stimulus;
					F: motor activity;
					F: actin binding;
					F: protein kinase activity;
					F: ATP binding;
					C: cytoplasm;
					C: myosin complex
Chr31:16977384	*NUTM2E*	5.71 × 10^−^^22^	100		
Chr19:11148798	*PREX1*	1.29 × 10^−^^21^	88.94	P: GO:0035556; F: GO:0005085	P: intracellular signal transduction;
					F: guanyl-nucleotide exchange factor activity
ChrX:113249264	LINE-1 retro transposable element ORF2 protein	3.24 × 10^−^^16^	84.22		
Chr33:4467956	hypothetical protein CB1_000568025	8.33 × 10^−^^15^	91.67		
ChrX:60452363	hypothetical protein HJG63_011487	8.67 × 10^−^^12^	72.06		
Chr12:5711	reverse transcriptase family protein	3.45 × 10^−^^8^	70.44		
Chr18:29830420	DUF1725 domain-containing protein	9.72 × 10^−^^7^	67.49		
Chr11:72060113	*KCNK18*	5.06 × 10^−^^6^	66.67		
Chr1:63040834	*POLR2A*	1.03 × 10^−^^4^	93.09		
Chr1:63040824	*POLR2A*	1.08 × 10^−^^4^	88.89		
Chr11:72356436	hypothetical protein HJG59_007818	2.44 × 10^−^^4^	50		

## Data Availability

The datasets generated during the current study are available in the [dryad] repository, https://doi.org/10.5061/dryad.n2z34tmwf; accessed on 10 January 2022.
